# Re-evaluating serum angiotensin-converting enzyme in sarcoidosis

**DOI:** 10.3389/fimmu.2023.950095

**Published:** 2023-10-05

**Authors:** Shi-yue Zheng, Xin Du, Jian-zeng Dong

**Affiliations:** ^1^ Department of Cardiology, Beijing Anzhen Hospital, Capital Medical University, Beijing, China; ^2^ Department of Cardiology, The First Affiliated Hospital of Zhengzhou University, Zhengzhou, Henan, China

**Keywords:** sarcoidosis, ACE, gene polymorphism, sensitivity, specificity

## Abstract

Sarcoidosis is a systemic inflammatory disease of unknown etiology, which mainly affects the lungs and lymph nodes, as well as extrapulmonary organs. Its incidence, and prevalence rate, and disease course largely vary with regions and populations globally. The clinical manifestations of sarcoidosis depend on the affected organs and the degree of severity, and the diagnosis is mainly based on serum biomarkers, radiographic, magnetic resonance, or positron emission tomography imaging, and pathological biopsy. Noncaseating granulomas composing T cells, macrophages, epithelioid cells, and giant cells, were observed in a pathological biopsy, which was the characteristic pathological manifestation of sarcoidosis. Angiotensin-converting enzyme (ACE) was first found in the renin–angiotensin–aldosterone system. Its main function is to convert angiotensin I (Ang I) into Ang II, which plays an important role in regulating blood pressure. Also, an ACE insertion/deletion polymorphism exists in the human genome, which is involved in the occurrence and development of many diseases, including hypertension, heart failure, and sarcoidosis. The serum ACE level, most commonly used as a biomarker in diagnosing sarcoidosis, in patients with sarcoidosis increases. because of epithelioid cells and giant cells of sarcoid granuloma expressing ACE. Thus, it serves as the most commonly used biomarker in the diagnosis of sarcoidosis and also aids in analyzing its therapeutic effect and prognosis in patients with sarcoidosis.

## Introduction

Sarcoidosis is a multisystem inflammatory disorder disease, mainly affecting the lungs and lymph nodes, as well as extrapulmonary organs, such as the skin, heart, eye, and nervous system (1, 2). It may occur at all ages, with a peak incidence rate at the age of 30–50 years in men and 50–60 years in women (3). The incidence and prevalence of sarcoidosis significantly differ with regions and populations worldwide. This variation is based mainly on race, age, and sex. A recent epidemiological study of sarcoidosis showed that Sweden has the highest incidence and prevalence of sarcoidosis (11.5 and 160 per 100,000 inhabitants, respectively), while it is rare in areas such as Belgium and South Korea (4). Furthermore, race is an essential factor in estimating morbidity and mortality of sarcoidosis. Compared with whites, black patients, especially females, experience more severe phenotypes, more organ involvement, with higher hospitalization and mortality rates (5). Other race-related factors, including environmental exposures, may increase the burden of sarcoidosis (6–8). Overall, the mortality rate of sarcoidosis during the 5-year follow-up is approximately 7%, of which more than 60% of sarcoidosis deaths are due to pulmonary involvement worldwide, while 85% are due to cardiac involvement in Japan (9, 10). Sarcoidosis has a variety of clinical manifestations based on the activation and migration of T cells and macrophages to the affected organs, organ involvement, and its severity. Most sarcoidosis inflammation subsides on its own, and only 10%–30% of patients develop chronic sarcoidosis, eventually leading to the fibrosis of the affected organs, which may be affected by the interaction between genes and the environment (2, 4, 11).

## Sarcoidosis management

Diagnosing sarcoidosis is difficult due to its slow onset and atypical clinical manifestations, which is mainly based on serological, imaging and pathological biopsies, including noncaseating granulomas and excluding nodular reactions to other granulomatous diseases such as tuberculosis, lymphoma, cancer, and other idiopathic granulomas. The most commonly used serum biomarker in sarcoidosis is ACE, with high specificity (12). In addition to ACE, gamma globulin and lysozyme also have high specificity, while sIL-2R, CRP, and chitotriosidase are more sensitive (1, 13). Although sarcoidosis most usually infiltrates lungs and bilateral hilar lymphadenopathy, approximately 30% of patients have extrapulmonary sarcoidosis as the initial manifestation. During follow-up, more than 90% of patients had lung involvement, 50% had extrathoracic involvement, and 2% had isolated extrathoracic involvement.

The level of serum biomarkers and corresponding imaging examination are different with the different organs involved in sarcoidosis. Furthermore, the clinical diagnosis of sarcoidosis must be confirmed by histological biopsy, that is, non-caseous nodular granuloma composed of T cells, macrophages, epithelioid cells and giant cells. However, the diagnosis of granuloma is insufficient without consistent radiological findings (14). As the clinical manifestations of sarcoidosis are highly variable, the decision on whether and when to treat sarcoidosis depends on the affected organs and the extent to impairment of patients’ quality of life (3). The cornerstone of sarcoidosis treatment is immunosuppressive therapy. Corticosteroid prednisone, immunosuppressant methotrexate (MTX) or azathioprine, or biological agent infliximab should be given when sarcoidosis has obvious pulmonary symptoms or affects extrapulmonary organs, including heart, eye, skin, and nervous system (15). Serum biomarkers change with the progress and the improvement of sarcoidosis, so judging the trend of serum biomarkers, such as ACE, is helpful for us to further understand the progress of sarcoidosis, and even make timely therapy before biopsy diagnosis.

## Angiotensin-converting enzyme

ACE (also known as peptidyl-dipeptidase A; CD143; EC3.4.15.1) was first found in the renin–angiotensin–aldosterone system (RAAS), which was earlier known as hypertension invertase (16, 17). Normally, ACE mRNA can be detected in systemic tissues, but the highest expression is on the surface of pulmonary capillaries and renal epithelial cells, which increases with age (18, 19). Human ACE is a polypeptide composed of 1306 amino acids and its molecular weight is about 146.6 kDa (20). ACE is a zinc-dependent dicarboxypeptidase that has two catalytic domains: C- terminal and N- terminal. These two domains contain the same zinc–binding motif HEXXH (where X can be any amino acid) and downstream E residues. The C-terminal has an angiotensin (Ang) transition site that helps in regulating blood pressure, while the N- terminal shows minimal effect. The outside of the active site of ACE is composed of two chloride ions that are significant in maintaining the structure and catalytic activity of ACE (21).

Humans have two forms of ACE: somatic ACE (sACE) and testis ACE [tACE, also known as germinal ACE (gACE)]. sACE is composed of two domains: C-terminal and N-terminal, while tACE only has C-terminal (22). sACE exists in a variety of tissues, while tACE is expressed only in sperm cells and mature spermatozoa in the testis (23). ACE, as a peptidase, can decompose the substrate peptides. It can remove the carboxyl-terminal dipeptides of decapeptide angiotensin I (Ang I) and convert it into octapeptide Ang II, which plays an important role in the RAAS (24). ACE can also decompose other substrate peptides. Besides Ang I, sACE substrate peptides include N-acetyl-Asp-Lys-Pro, substance P, enkephalin, luteinizing hormone–releasing hormone, kinin, amyloid-beta peptide, and neurotensin (25). tACE can hydrolyze most of the substrates of sACE, except Ang I (26).

## ACE gene polymorphism

ACE is encoded by a single gene located on chromosome 17q23, comprising 26 exons and 25 introns, with a length of 21 kb (27). sACE, which is transcribed from exons 1–12 and 14–26, is long with a molecular weight of about 170 kDa. tACE is transcribed from exon 13–26 with a shorter length and a molecular weight of about 100 kDa (28). An insertion/deletion (I/D) polymorphism is found in intron 16 of the ACE gene, and the length of I/D fragment is 287 bp (29). Therefore, ACE has three main genotypes: DD, ID, and II. The 287-bp fragment in the ACE gene was a member of the AluYa5 subfamily of Alu elements and encoded an RNA molecule that could regulate the level of ACE mRNA (30). Hence, the gene polymorphism of ACE affects the serum ACE level, in which DD genotype has the highest and the II genotype has the lowest value (**Table 1**) (32).

**Table 1:** Serum ACE levels of different genotypes of ACE (29, 31).

Serum ACE level	Genotypes			
	II	ID	DD	
Normal	9.0–12.5	10.5–17.1	11.9–22.5	IU/L†
	3.76–11.25	5.22–11.59	7.19–14.84	U/L‡
Sarcoidosis	13.5–29.3	16.7–31.1	19.8–34.8	IU/L†

ACE, Angiotensin-converting enzyme.

†Serum ACE level was measured by a colorimetric method using p-hydroxyhippuryl-l-histidyl-l-leucine as the substrate.

‡Optimized Serum ACE level was measured by internally quenched fluorescent substrate, Abz-FRK(Dnp)P-OH.

## Sarcoidosis and ACE

Sarcoidosis is also a polygenic disease without specific causative pathogenic genes, and its pathogenesis is results from granulomatous inflammation triggered by the interaction between genes and the environment (11). The epithelioid and giant cells that compose granuloma can express ACE, increasing the level of serum ACE in patients with sarcoidosis (33). Serum ACE can also reflect the severity of sarcoidosis granulomas and can be used to monitor disease activity and treatment response, with high specificity especially when it’s twice as high as the normal limit (34, 35). About 30%–80% of patients with sarcoidosis have an elevated serum ACE levels, with a sensitivity of 22%–86% and a specificity of 54%–95% (**Table 2**) (1).

**Table 2:** Sensitivity and specificity of serum ACE in different types of sarcoidosis.

	Sensitivity (%)	Specificity (%)	PPV (%)	NPV (%)
Sarcoidosis				
serum ACE	22–86	54–95	–	–
Ocular sarcoidosis (*n* = 249) (36)				
serum ACE ≥68 U/L	30	85	26	87
serum ACE ≥51 U/L	54	70	24	90
serum ACE ≥68 U/L + chest radiograph	70	79	42	92
serum ACE ≥51 U/L + chest radiograph	82	64	33	94
Ocular sarcoidosis (*n* = 37) (37)				
serum ACE	27	96.6	90.9	51.7
Ocular sarcoidosis (*n* = 22) (38)				
serum ACE	73	83	–	–
serum ACE + positive ^67^GA scanning	100	73	–	–
Ocular sarcoidosis (*n* = 79) (39)				
serum ACE	37.7	97.5	–	–
Cardiac sarcoidosis (*n* = 94) (40)				
serum ACE ≥13.5 IU/L + positive EMB	56.8	54.4	44.7	66.0
serum ACE ≥13.5 IU/L + positive EMB + LVEF <37%	37.8	91.2	73.7	69.3

ACE, Angiotensin-converting enzyme; EMB, endomyocardial biopsy; LVEF, left ventricular ejection fraction; NPV, negative predictive value; PPV, positive predictive value.

The serum ACE activity is affected by the site of sarcoidosis lesions and the ACE gene I/D polymorphism. Significant differences in the serum ACE level may be found among different ethnic groups, and the normal range of serum ACE is defined according to different genotypes regardless of their level of involvement in human organs (29, 41). ACE gene I/D polymorphism is not the main genetic cause of sarcoidosis, although ACE is related to its pathogenesis and more likely changes its progression (42). Kieszko et al. (43) reported that the incidence of sarcoidosis was higher among individuals living in rural areas and carriers of specific ACE genotypes. It might be because of the change in environment, urbanization, and pollution in rural areas, indicating the pathogenesis of sarcoidosis via interaction between genes and environment. Papadopoulos et al. (44) confirmed that the frequency of ACE DD genotype in patients with sarcoidosis having autoimmune manifestations increased significantly with the increasing level of serum ACE. It is speculated that the homozygous D allele of ACE may contribute to the autoimmune susceptibility of sarcoidosis through the elevated serum ACE level. Besides, ACE gene mutation can also increase the serum ACE level by about 5–20 times, which may be achieved by eliminating of the transmembrane anchor and a putative glycosylation site in the ACE gene (45). It demonstrates that serum ACE vary widely between genotypes, suggesting that clinicians should incorporate exome sequencing in the diagnosis of sarcoidosis to avoid unnecessary invasive biopsies. The clinical study conducted by Csongradi et al. (31) reported that the detection of serum ACE activity combined with the ACE I/D genotype could improve the diagnosis of sarcoidosis with 42.5% sensitivity, 100% specificity, 100% positive predictive value, and 32.4% negative predictive value.

Corticosteroid prednisone, immunosuppressant MTX or azathioprine, or biological agent infliximab should be given when sarcoidosis has obvious pulmonary symptoms or affects extrapulmonary organs, including heart, eye, skin, and nervous system (15). After treatment with the aforementioned drugs, the serum ACE level decreased to normal with the improvement in the condition, and increased again after sarcoidosis recurrence. However, Di Francesco et al. (46) confirmed that serum ACE could not distinguish between the disease state prior to and after the corticosteroid therapy of patients with sarcoidosis, although serum ACE level in patients could help detect both the active sarcoidosis and sarcoidosis in remission/under treatment. Infliximab, the first monoclonal tumor necrosis factor antibody for human treatment, is an effective third-line drug used for the therapy of severe sarcoidosis. Croft et al. (47) reported that the clinical symptoms of patients with refractory sarcoidosis treated with infliximab were relieved, with the serum ACE level reduced in all cases. Inflectra, which is biosimilar to infliximab, can also be used in the therapy of severe sarcoidosis, with clinical symptoms improved and the serum ACE levels reduced after 26 weeks of Inflectra treatment (48). It has shown that patients who have been treated with corticosteroids since diagnosis have a 5-year survival rate of at least 75%, compared with 10% for untreated patients. Therefore, understanding the change of serum ACE levels will help us to carry out immunosuppressive therapy earlier before the diagnosis of sarcoidosis, which is very likely to further improve the survival rate of patients (49).

## Pulmonary sarcoidosis

Although sarcoidosis can involve multiple organs, the most frequently involved are the lungs and lymph nodes. Sarcoidosis can be divided into five stages based on the chest X-rays: radiograph showing the normal chest (stage 0), nodal enlargement only (stage I), nodal enlargement and parenchymal opacity (stage II), parenchymal opacity without adenopathy or evidence of fibrosis (stage III), and lung fibrosis (stage IV). Radiographs of about 5%–15% of the patients showed stage 0, 45%–65% stage I, 30%–40% stage II,10%–15% stage III, and 5% stage IV (50). The natural remission rate of sarcoidosis was recorded in 60%–90% of patients in stage I, 40%–70% in stage II, and 10%–20% in stage III (34). More than 90% of patients with sarcoidosis have the chest affected, but up to 50% of patients with sarcoidosis have never shown clinical symptoms, while approximately one third experience the chronic stage. Compared with isolated thoracic lymphadenopathy, patients with parenchymal pulmonary sarcoidosis can progress to pulmonary fibrosis, usually with a high recurrence and mortality rate (35, 51). Although it has been found that several serum biomarkers may increase in the pathogenesis of sarcoidosis, reliable biomarkers have not been found in clinic. Serum ACE is a commonly used biomarker in diagnosis of sarcoidosis, mainly produced by activated alveolar macrophages. It is related to the granulomatous load and radiological stages II and III, and its increase was observed in more than 75% of untreated patients, which is an indirect evidence of persistent sarcoidosis activity (1, 52). A cross-sectional study in China showed that the serum ACE level in patients with pulmonary sarcoidosis concomitant extrapulmonary involvement was higher than that in patients with isolated pulmonary sarcoidosis, and was related to extrapulmonary organ involvement and overall disease activity (53). At present, no association exists between the ACE gene I/D polymorphism and degree of severity, fibrosis, and progression of pulmonary sarcoidosis (54). In addition to chest X-rays, chest computed tomograph, 18F-fluorodeoxyglucose positron emission tomography, and bronchoscopy combined with biopsy, including transbronchial aspiration, and endobronchial biopsy also have high diagnostic value (55). Treatment is not recommended clinically due to the spontaneously resolution can be observed in asymptomatic patients with stage I or II sarcoidosis. Corticosteroids should be considered for patients with obvious symptoms or progressive stage II or III pulmonary diseases or severe extrapulmonary diseases (56, 57). Studies showed that 50%–90% of patients with sarcoidosis having pulmonary symptoms responded well to corticosteroids (58). The serum ACE activity started decreasing within 2 weeks of using prednisone and remained normal within 5 months of using low-dose prednisone maintenance therapy (**Table 3**). However, 20%–74% of patients relapsed after stopping therapy, resulting in an increase in the serum ACE level (59, 60). Hence, the serum ACE level can be used to monitor whether sarcoidosis is successfully treated. However, there is no evidence that oral corticosteroids can improve patients’ long-term outcomes, including readmission and mortality rate. Patients who are ineffective or intolerant to corticosteroids can be treated with MTX, leflunomide, azathioprine, and biological agents (61). The level of serum ACE decreased significantly and the pulmonary function of patients improved obviously 6 months after treatment with MTX. Also, the high baseline level of serum ACE correlated with the improvement in pulmonary function after treatment (62). Continuous measurement of the serum ACE level can help in monitoring the therapeutic effect in patients with sarcoidosis.

**Table 3:** Changes in ACE level during the treatment of different types of sarcoidosis.

Sarcoidosis	Treatment	Time	ACE level
Pulmonary sarcoidosis	Prednisone	2 weeks	Serum ACE decrease
	MTX	6 months	
Cutaneous sarcoidosis	Thalidomide	3 months	Serum ACE decrease
	Melatonin	12 months	
Ocular sarcoidosis	Corticosteroid eye drops	20 months	Serum ACE increase
Neurosarcoidosis	Corticosteroids or corticosteroids combined with MTX	Unknown	ACE decrease in CSF
Systemic sarcoidosis	Inflectra	26 weeks	Serum ACE decrease

ACE, Angiotensin-converting enzyme; CSF, cerebrospinal fluid; MTX, methotrexate.

## Cutaneous sarcoidosis

Skin is the second most common organ affected in sarcoidosis impacting about one-third of patients. This isolated cutaneous sarcoidosis has multiple erythemas, plaques, or subcutaneous nodules as the main manifestations. The histological examination can result in the absence of characteristic nodular granulomas, which refers to non-specific cutaneous sarcoidosis. Nodular erythema is the most common nonspecific lesion, occurring in up to 25% of patients (63). The treatment of patients with cutaneous sarcoidosis can be divided into topical and systemic therapies. Corticosteroids are the most commonly used agents in topical and systemic therapies; systemic therapy also includes tetracycline antibiotics, phosphodiesterase inhibitors, and MTX (64). The change in the serum ACE level after corticosteroid treatment of sarcoidosis has been reported, but whether the topical application of corticosteroids can also change the level of serum ACE is yet to be studied. The patients with cutaneous sarcoidosis experiencing steroid resistance or contraindications can be treated with thalidomide (an immunomodulatory drug). The serum ACE level begins to decrease gradually after treatment with thalidomide, drops to normal in the third month, and then continues to decline. Hence, the decrease in the serum ACE level has a significant correlation with the course of the disease (65). Pignone et al. (66) found that melatonin, which is also an immunomodulatory drug, could be used to treat cutaneous sarcoidosis, besides the thalidomide treatment. The serum ACE level gradually decreased to normal in about 12 months of melatonin treatment, and in about 24 months, the skin lesions completely disappeared and the pulmonary function returned to normal; no recurrence of sarcoidosis was observed during the treatment. Kaura et al. (67) reported a case and showed that ACEIs, especially benazepril, might also be an effective drug for treating cutaneous and lymph-node sarcoidosis. ACEIs can reduce the serum ACE level, so the serum ACE level should not be used to diagnose and evaluate sarcoidosis in patients treated with ACEIs. However, the effects of all ACEIs on the serum ACE level are not similar. For example, zofenopril does not affect the serum ACE level compared with ramipril, enalapril, and perindopril (68). However, whether benazepril has any effect on the serum ACE level remains to be confirmed.

## Cardiac sarcoidosis

Clinically, about 3%–39% of patients with systemic sarcoidosis have cardiac involvement, while the incidence of isolated CS in patients with CS is about 27%–54% (69). About 5%–8% of patients with CS have clinical manifestations, including conduction abnormalities, ventricular arrhythmias, and heart failure (HF), while 20%–25% of patients have asymptomatic cardiac involvement (63, 70). The CS diagnosis is mainly based on the combination of serological markers, such as serum ACE, imaging examinations, including cardiac magnetic resonance imaging and positron emission tomography, and endomyocardial biopsy (EMB) following the Japanese Circulation Society (JCS) protocols (71, 72). The sensitivity and specificity of serum ACE in diagnosing CS are low. A study of 172 patients with sarcoidosis showed that the serum ACE levels in patients with isolated CS were lower than the levels in those with non-isolated CS, implying that higher serum ACE levels were related to systemic lesions (73). The proportion of cardiac granuloma was about 46.9% based on the autopsy reports, while the proportion of non-caseating granulomas found via EMB was about 20%–30% (63, 74). Only about 20% of patients with CS show positive EMB (71). Although the aforementioned diagnostic methods have low specificity and sensitivity, a combination of these diagnostic methods can improve the specificity and sensitivity. The sensitivity and specificity of serum ACE increased by 56.8% and 54.4%, respectively, in patients with positive EMB. If a combined left ventricular ejection fraction reduced at the same time, the specificity of serum ACE further increased to 91.2% and the sensitivity was 37.8% (40). Although the treatment of CS has not been standardized, corticosteroids are still used to control inflammation and prevent cardiac fibrosis in patients with CS as the first-line drug of choice (75, 76). When patients with CS develop arrhythmias or HF, immunosuppressants and cardiac assist devices can reduce the long-term mortality in patients (77). How the serum ACE level changes during the treatment of CS is yet to be reported. When patients with CS have HF or hypertension, ACEIs cannot be used to measure the SACE level to evaluate the patients’ condition and therapeutic effect in those treated with ACEIs. Although the serum ACE activity can be inhibited by ACEIs, the level of ACE protein is not affected by these drugs. Therefore, the detection of ACE protein activity is more suitable for the diagnosis and prognosis of sarcoidosis than the detection of ACE activity (78).

## Other forms of sarcoidosis

Ocular sarcoidosis (OS) is reported in about 25%–50% of patients with systemic sarcoidosis. It is mainly characterized by anterior uveitis involving young patients or total uveitis in middle-aged and elderly patients (74). The sensitivity and specificity of serum ACE in patients with OS are 27%–73% and 70%–97.5%, respectively (36–39). For patients with OS whose whole-body gallium (^67^Ga) scanning was positive, the sensitivity of serum ACE increased to 100%, and the specificity was 73% (38). The serum ACE level was significantly higher in patients with OS treated with corticosteroid eye drops for 20 months than that in non-treated group, and the enlarged lymph nodes in the chest radiograph suggested that the local corticosteroid treatment might affect the clinical course of pulmonary sarcoidosis (79). About 84%–94% cases of patients have systemic sarcoidosis, while isolated neurosarcoidosis is extremely rare, with a prevalence rate of about 1%–17%. The specificity of ACE in the cerebrospinal fluid (CSF) is 67.3%–95% and the sensitivity is 0%–66.7% (63). The clinical outcome of patients with sarcoidosis can be improved with the use of corticosteroids and immunosuppressants (80). In patients with neurosarcoidosis, the serum ACE level decreased significantly after treatment with corticosteroids or corticosteroids combined with MTX, but the decrease in the ACE level in CSF was not as significant as that of serum ACE (81).

## Conclusions

The serum ACE level increased in patients with sarcoidosis, dropped to normal with improvement in condition during treatment, and increased again after the recurrence of sarcoidosis. Serum ACE level is related to the systemic lesions of sarcoidosis, which is lower in patients with isolated sarcoidosis than in those with non-isolated sarcoidosis. In addition, the level of serum ACE is also affected by ACE gene I/D polymorphism, so clinically, ACE genotypes should be detected first for patients with elevated serum ACE level, and the relationship between elevated serum ACE level and sarcoidosis can be determined according to the ACE genotypes. Most laboratories detect the level of serum ACE, which also increased in other diseases such as tuberculosis and tumor, besides sarcoidosis. The serum ACE level was also affected by ACE gene I/D polymorphism and use of drugs such as ACEIs, while the level of ACE protein did not change. We should take ACE protein level as a serological marker for the diagnosis of sarcoidosis, which has more clinical significance. At the same time, we should clarify the changes of ACE protein level in different types and different stages of sarcoidosis to help us diagnose sarcoidosis and understand its pathogenesis better. Therefore, re-evaluating the ACE level in sarcoidosis enhances the understanding of sarcoidosis in clinic.

## Author contributions

All authors listed have made a substantial, direct, and intellectual contribution to the work, and approved it for publication.
